# *Doublecortin* Knockout Mice Show Normal Hippocampal-Dependent Memory Despite CA3 Lamination Defects

**DOI:** 10.1371/journal.pone.0074992

**Published:** 2013-09-20

**Authors:** Johanne Germain, Elodie Bruel-Jungerman, Gael Grannec, Cécile Denis, Gabriel Lepousez, Bruno Giros, Fiona Francis, Marika Nosten-Bertrand

**Affiliations:** 1 INSERM UMRS 952, Paris, France; 2 CNRS UMR 7224, Paris, France; 3 UPMC, Paris, France; 4 Université Paris Descartes, Paris, France; 5 INSERM UMR-S 839, Paris, France; 6 Institut du Fer à Moulin, Paris, France; 7 Unité Perception et Mémoire, Institut Pasteur, Paris, France; 8 Douglas Hospital Research Center, Department of Psychiatry, McGill University, Montreal, Canada; University of Queensland, Australia

## Abstract

Mutations in the human X-linked doublecortin gene (*DCX*) cause major neocortical disorganization associated with severe intellectual disability and intractable epilepsy. Although *Dcx* knockout (KO) mice exhibit normal isocortical development and architecture, they show lamination defects of the hippocampal pyramidal cell layer largely restricted to the CA3 region. *Dcx*-KO mice also exhibit interneuron abnormalities. As well as the interest of testing their general neurocognitive profile, *Dcx*-KO mice also provide a relatively unique model to assess the effects of a disorganized CA3 region on learning and memory. Based on its prominent anatomical and physiological features, the CA3 region is believed to contribute to rapid encoding of novel information, formation and storage of arbitrary associations, novelty detection, and short-term memory. We report here that *Dcx*-KO adult males exhibit remarkably preserved hippocampal- and CA3-dependant cognitive processes using a large battery of classical hippocampus related tests such as the Barnes maze, contextual fear conditioning, paired associate learning and object recognition. In addition, we show that hippocampal adult neurogenesis, in terms of proliferation, survival and differentiation of granule cells, is also remarkably preserved in *Dcx*-KO mice. In contrast, following social deprivation, *Dcx*-KO mice exhibit impaired social interaction and reduced aggressive behaviors. In addition, *Dcx*-KO mice show reduced behavioral lateralization. The *Dcx*-KO model thus reinforces the association of neuropsychiatric behavioral impairments with mouse models of intellectual disability.

## Introduction

Cortical malformations are associated with epileptic syndromes and severe intellectual disability. Type I lissencephaly, a cortical lamination disorder, is characterized by a smooth brain surface and a thickened and severely disorganized neocortex, as well as abnormally formed hippocampi [1]. X-linked *Doublecortin* (*DCX*), *LIS*1, *reelin* (*RELN*) and *alpha tubulin* 1A (*TUBA1A*) genes are mutated in these disorders [2], [3], [4], [5], [6] and thought to play a role in neuronal migration.

The physiopathological consequences of such cortical lamination defects and their links with epileptiform activities and intellectual disability are not well understood. Efforts have hence been made to generate and study mouse models mutant for the above-mentioned genes. One feature common to these models is their hippocampal lamination defects, which contributed to the identification of *TUBA1A* as a lissencephaly gene [4]. Heterozygote *Lis*1 and *Tuba1a* mutant mice show only subtle isocortical layering abnormalities [4], [7]. However, in both models, CA1 and CA3 pyramidal cells are disorganized [4], [7], [8], [9]. *Reeler* mice show severe disorganization of the hippocampus as well as the isocortex [10]. *Dcx*-KO mice on the other hand show no lamination defects in the isocortex and a cellular disorganization largely restricted to the CA3 region of the hippocampus [1], [11], [12].

We previously showed that *Dcx*-KO mice exhibit spontaneous convulsive seizures that are initiated in the hippocampus [13]. These data were suggestive of abnormal synaptic transmission involving heterotopic cells in the CA3 region. Further studies showed that CA3 pyramidal cells have simplified dendritic arbors, mossy fiber connection abnormalities, and are more excitable than WT CA3 cells, which may contribute to the susceptibility to epilepsy in this model [14]. Thus clear consequences of the CA3 lamination defect are observed at the level of excitability.

With only restricted anatomical modifications, *Dcx-*KO mice provide a pertinent model to explore and evaluate hippocampal-dependent learning and memory processes. The anatomical and physiological characteristics of the CA3 region – on which converging inputs from a) the entorhinal cortex via the perforant path, b) the dentate gyrus via mossy fibers, and c) its own inputs via the recurrent collaterals - inspired many theoretical models to assign specific cognitive processes to this field. Amongst these, the recurrent collateral circuitry, by which the CA3 pyramidal cells make excitatory synaptic contacts with each other, could serve a critical role in tasks involving rapid contextual representation, novelty detection, and one-trial short-term memory, that all require arbitrary and conjunctive associations [15], [16], [17], [18].

The aim of the present study was thus to explore in detail classical hippocampus-and CA3-dependent learning and memory tasks in *Dcx*-KO mice. We also selected behavioral tests commonly used to assess models of intellectual disability, such as social interaction and behavioral lateralization.

## Results

### Adult *Dcx-KO* Mice have a Slower Adult Body Growth Rate than WT Mice

We previously showed that *Dcx*-KO mice exhibit normal postnatal growth rate until weaning, but a significantly lower body weight at 4 months of age [13]. In a much larger cohort, we confirm here that *Dcx*-KO mice displayed a significantly slower adult growth rate, exhibiting 3% to 7% reduction in body weight compared to their WT littermates between 1 to 7 months of age (Fig. S1).

We also previously reported a novelty-induced hyperactivity in actimeter cages [13]. This hyperactivity was not systematically observed, but rather dependent on the experimental context. Indeed, *Dcx*-KO mice exhibited the same level of activity compared to WT mice (Fig. S2), in the open field (social interaction) and the Y-maze (odor discrimination).

### Hippocampal Dependent Spatial Navigation is Unimpaired in *Dcx-KO* Mice

**Figure 1:**
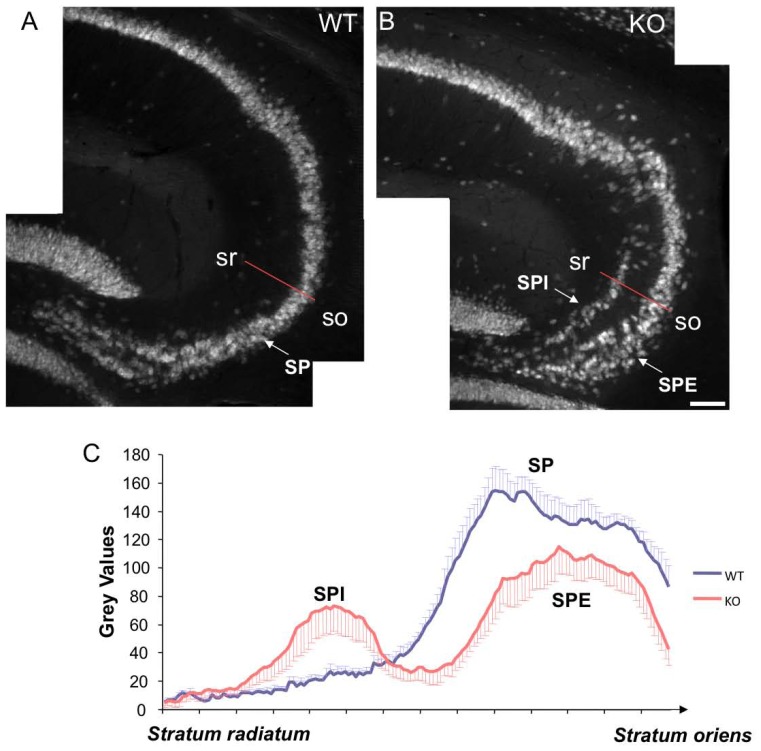
Abnormal hippocampal lamination in the *Dcx-*KO mice. (A–B) NeuN labeled sagittal sections of adult hippocampus from a control (A) and a *Dcx*-KO (B) mouse showing a largely normal CA1 region but a disorganized CA3 region. This disorganization is characterized by two distinct pyramidal cell layers (termed *stratum pyramidale internal*, SPI, and *stratum pyramidale external,* SPE). (C) Histogram showing the distribution of pixel intensities along the red lines as indicated in A and B, across the CA3 region and extending from the *stratum radiatum* (sr) towards the *stratum oriens* (so) from both control (blue) and KO (red) mice. The distribution of these grey values clearly shows a double peak in the KOs corresponding to SPI and SPE respectively, in comparison to a single peak (corresponding to the *stratum pyramidal*, SP) in the WTs. Scale bar 60 µm.

**Figure 2:**
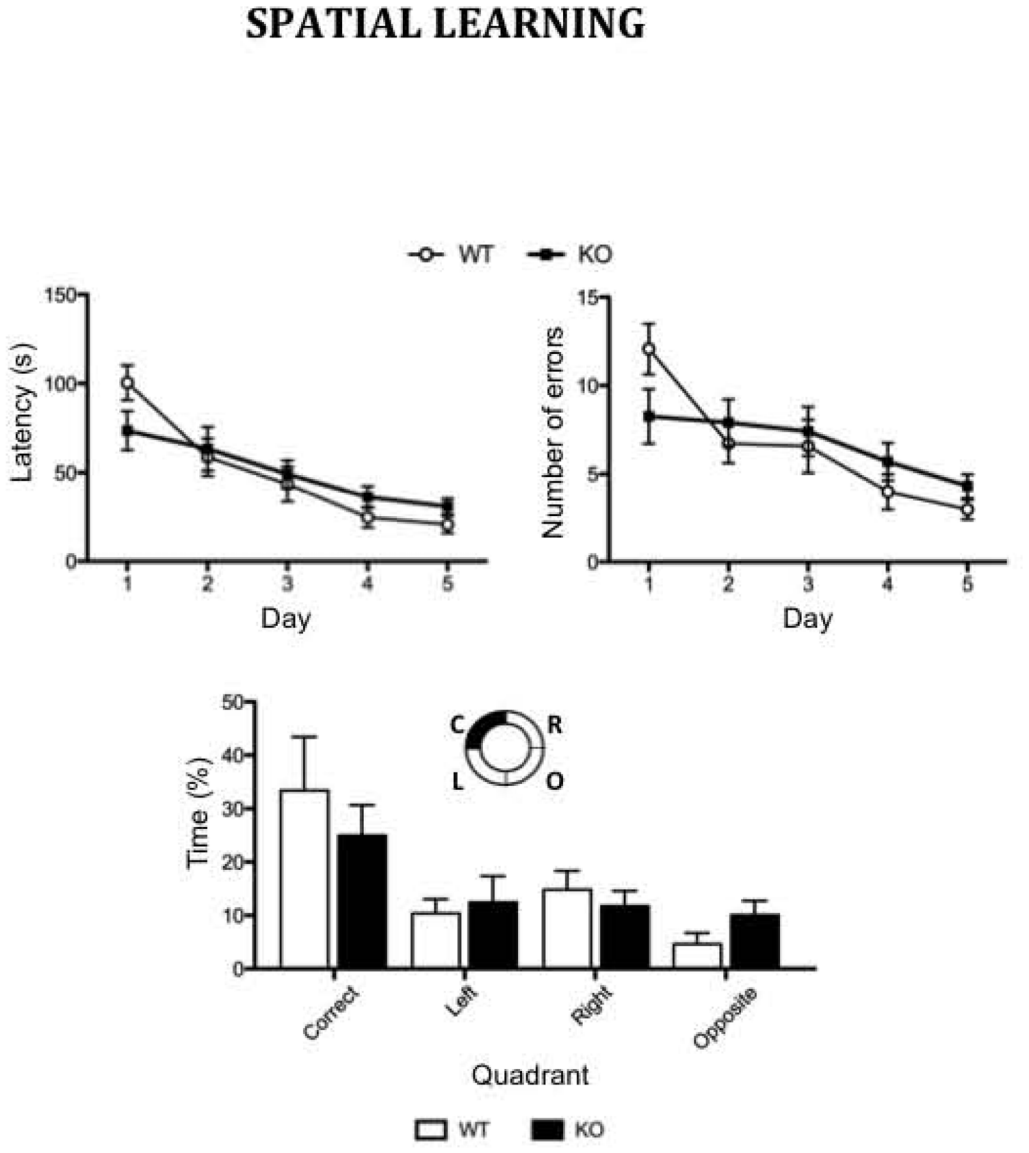
Normal spatial learning and memory in the *Dcx-*KO mice. During acquisition trials in the Barnes maze, performances of *Dcx*-KO (n = 7) and WT (n = 8) male mice are expressed as latency (s) to escape the platform and number of errors. During the probe test, spatial strategy is expressed as percentage of time spent at the periphery of the maze, in the target, right, left, and opposite quadrants, as indicated in the schema. Values represent means +/− SEM.

As previously described [1], [11], [12], [13], [14], and as quantified here, a clear lamination defect in the CA3 region of the hippocampus is consistently observed in *Dcx*-KO mice, with pyramidal cells abnormally dispersed and forming two distinct layers (Fig. 1). Given the prominent role of the hippocampus in spatial representation of the environment, *Dcx*-KO mice were first tested for long-term spatial learning and memory in the Barnes circular maze. As *Dcx*-KO mice explored the maze at the same speed as their WT littermates (data not shown), latency to find the escape chamber was chosen as a learning variable. During the 5 days of training (Fig. 2), *Dcx*-KO mice learned the fixed position of the hidden tunnel at rates similar to their WT littermates as illustrated by a similar latency to escape (genotype, F_1,69_ = 0.034, p>0.05) and number of errors (genotype, F_1,69_ = 0.102, p>0.05) that decreased during the acquisition period for the two genotypes (day of training, F_4,69_ = 16.09, p<0.001 and F_4,69_ = 8.44, p<0.001, respectively). During the probe test, when the tunnel was removed, no significant difference in search strategy between the two groups of mice was detected (genotype, F_1,56_ = 0.08, p>0.05; Fig. 2). *Dcx*-KO mice, like their WT littermates, spent more time searching in the target quadrant (quadrants: F_3,56_ = 7.47, p<0.001), suggesting that despite a major hippocampal lamination phenotype, spatial navigation is unimpaired in *Dcx*-KO mice.

### Hippocampal and CA3-dependent Functions are Normal when Tested in the Fear-conditioning Paradigm

Hippocampal involvement in contextual representation has also been extensively investigated in Pavlovian fear conditioning paradigms [19], [20], [21], [22], [23], [24], [25].

**Figure 3:**
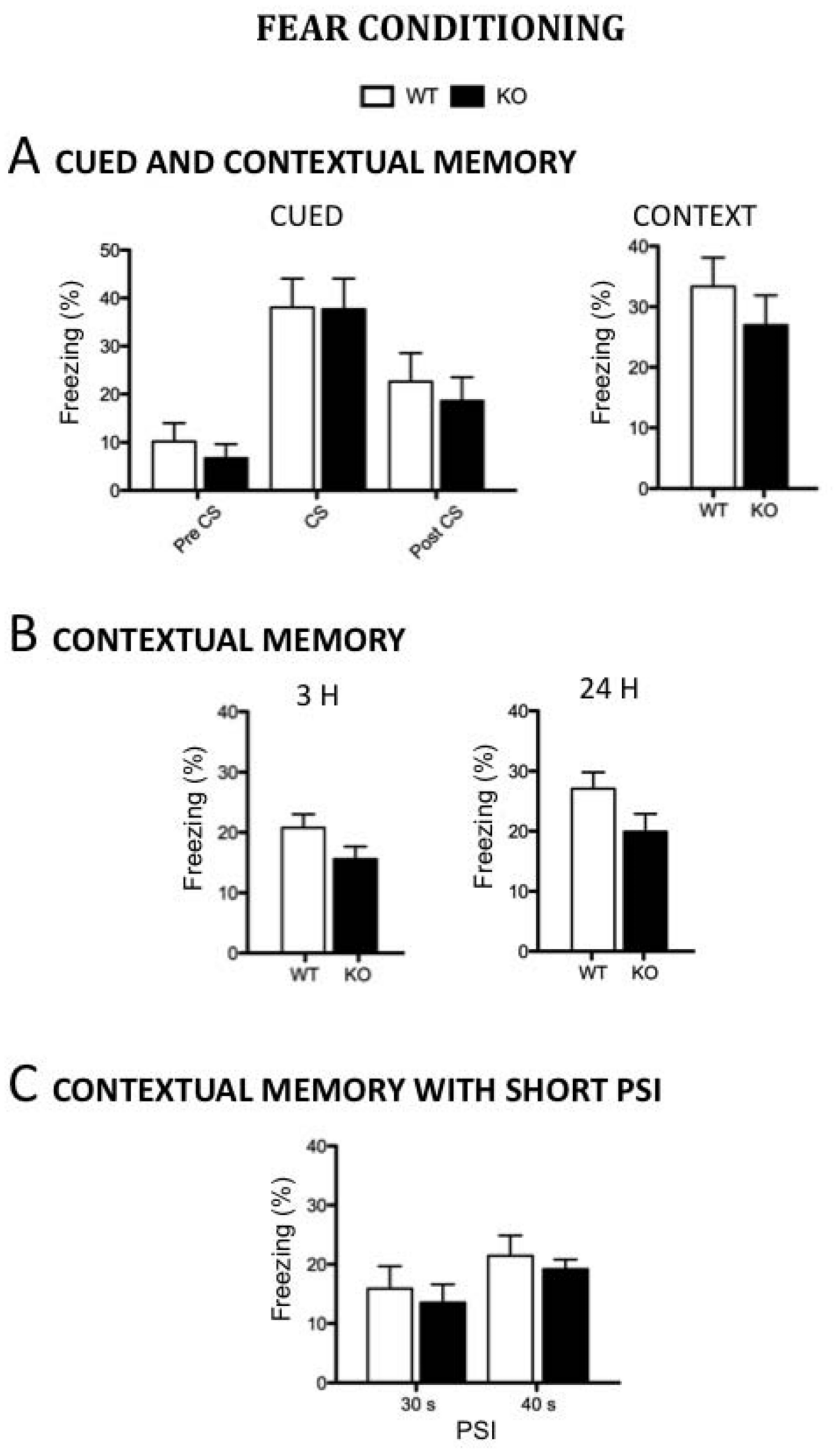
Normal fear conditioning in the *Dcx-*KO mice. A/Cued and contextual memory*;* percentage of time spent freezing to tone (left) and to context (right) 24h after training in two context-tone-shock pairing trials (n = 8–9 mice per group)*;* **B/Contextual** **memory** ***;*** percentage of time spent freezing to context 3 h (left) (n = 13 mice per group) and 24 h (right) after training in one context/shock pairing trial (n = 9–16 mice per group)*;* **C/Contextual** **memory** ***;*** percentage of time spent freezing following a short PSI (30 s: n = 6–8 mice per group*;* 40 s: n = 19–21 mice per group). Values represent means +/− SEM.

We first assessed both contextual and cued memory in *Dcx*-KO and WT mice using a protocol with two tone-shock pairings on the training day. Mice were tested 24h later for cued-conditioning to the tone in the novel environment. No significant difference was observed between genotypes in freezing, neither before (PreCS) nor after (PostCS) the tone (genotype, F_1,15_ = 0.63, p>0.05 and F_1,15_ = 0.261, p>0.05, respectively), and both WT and KO mice exhibited equal enhanced freezing during the 30 s exposure to the tone (genotype, F_1,15_ = 0.002, p>0.05), demonstrating normal cued memory (Fig. 3A). One hour later, the mice were tested for fear of the conditioning context. Again, both WT and KO mice exhibited similar enhanced freezing (genotype, F_1,15_ = 0.872, p>0.05) suggesting that contextual memory is spared in *Dcx*-KO mice.

Previous studies have shown that hippocampal damage does not fully prevent contextual memory, but rather slows down the rate of learning [26], [27]. Indeed, it has been shown that lesion of the hippocampus only impairs contextual fear memories when animals are exposed to a single, but not to multiple context-shock pairings [20], [28]. For instance, impairment of contextual memory was identified in conditional knockout mice with an NMDA receptor (NR) deletion restricted to CA3 pyramidal cells (CA3-NR1 KO mice) following one context-shock pairing, but was not revealed when animals were exposed to a protocol using multiple context-shock pairings [29], [30], suggesting that the CA3 region contributes speed to contextual processing. We thus set up a second protocol on independent groups of *Dcx*-KO and WT mice using only one context-shock pairing and no cue (Fig. 3B). Again, *Dcx*-KO mice exhibited similar enhanced freezing to the context as compared to their WT littermates, either tested 3 (genotype, F_1,24_ = 2.917, p>0.05) or 24 h (genotype, F_1,24_ = 2.69, p>0.05) after conditioning, supporting the fact that contextual memory is apparently intact in *Dcx*-KO mice.

Previous experiments have shown that little to no learning occurs when shock is presented immediately after animals are placed in the context (immediate shock deficit), and increasing the amount of context exposure before the shock alleviates this deficit [31], [32]. In addition, CA3-NR1 mutant mice exhibit contextual conditioning deficits at short, but not at longer placement-to-shock intervals (PSI), revealing a quantifiable contribution of the CA3 region to the rapid formation of contextual representation [29], [30]. We thus carried out a new fear conditioning protocol on independent groups of *Dcx*-KO and WT mice following a one-trial conditioning paradigm with a short PSI. Again, when tested 24 h later in the same context, both control and KO mice exhibited the same amount of freezing (30s PSI: genotype, F_1,23_ = 0.045, p>0.05; 40s PSI: genotype, F_1,69_ = 0.034, p>0.05), with less freezing for the shortest PSI of 30 s (Fig. 3C), indicating that, unlike CA3-NR1 mutants, the CA3 hippocampal lamination abnormalities in *Dcx*-KO mice do not impair the rapid encoding of new contextual information.

### Context Discrimination and Contingent Paired Associate Learning are Spared in *Dcx-KO* Mice

Computational models, and behavioral and electrophysiological data, suggest that the recurrent excitatory connections among CA3 cells form an auto-associative network that confers the CA3 region with highly specific cognitive functions. The recurrent collateral associative connections may enable rapid detection and encoding of novel information allowing the acquisition of contingent associations [15], [33], [34]. Indeed, in both rats and mice, the CA3 region is required for paired-associate learning [35], [36], as well as for novelty detection [37]. We thus tested *Dcx*-KO mice for context-odor paired associate learning using the protocol described by Rajji [36]. In a preliminary set of experiments, the mice were trained with only two scents, one scent rewarded in context 1 and the other scent rewarded in context 2. We did not detect any differences in performance between the two genotypes and all mice exhibited high performance as early as the first day of training (data not shown).

**Figure 4:**
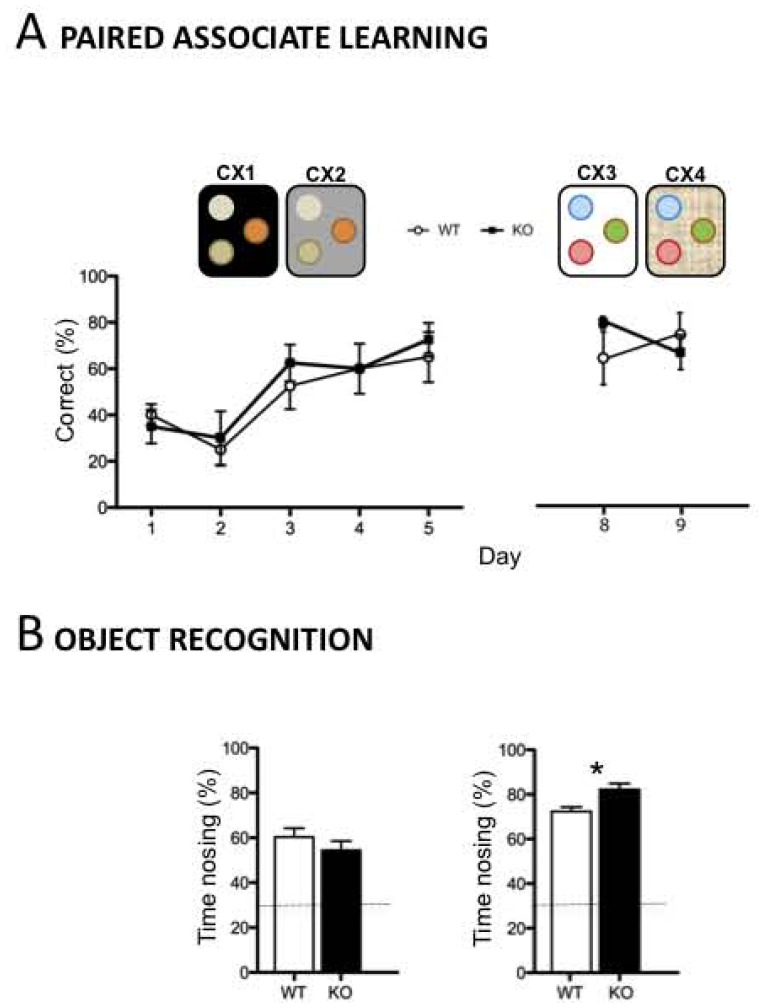
Normal contingent learning and novelty detection in the *Dcx-*KO mice. A/Paired-associate learning; performances of *Dcx*-KO and WT (n = 4 mice per genotype) male mice trained for five days in the paired-associate learning between two contexts (CX1 and CX2) and three olfactory cues, one baited (+) and two unbaited (−), followed by two days using novel contexts and novel odors. **B/Novelty detection;** Performances of *Dcx*-KO male mice expressed as the percentage of time spent exploring the displaced object (left) (n = 15 mice per genotype) or the novel object (right) (n = 10 mice per genotype) three minutes after the last habituation trials. The horizontal line indicates the by-chance exploration time for each of the three objects (33%). Values represent means +/− SEM.

In order to increase the difficulty (reducing the by chance finding), we set up a new protocol with animals exposed to two contexts, but three different scents, with only one scent rewarded (Fig. 4A). *Dcx*-KO like WT mice (genotype, F_1,40_ = 0.374, p>0.05), were able to learn such contingent associations with training (time: F_4,40_ = 7.14, p<0.001; genotype×time: F_4,40_ = 0.22, p>0.05). In addition, both *Dcx*-KO and WT mice showed high performances when exposed two days later to new contexts and new rewarded olfactory cues (Fig. 4A; genotype, F_1,46_ = 1.111, p>0.05); genotype×time, F_1,16_ = 1.884, p>0.05). All together, these results suggest that *Dcx* mutant mice, despite a disorganized CA3 region, are able to form rapid context-odor associations with novel stimuli to get a contingent reward.

Spatial and non-spatial novelty detection were then tested in *Dcx*-KO mice using object exploration in an open-field. Figure 4B shows that 3 min following the last habituation trials, *Dcx*-KO mice compared to their WT littermates spent the same amount of time exploring the displaced object (genotype, F_1,28_ = 1.11, p>0.05) and explored the novel object significantly more (genotype, F_1,18_ = 9.03, p>0.01). However, both KO and WT mice explored the displaced and a novel objects longer than the two other unchanged objects, indicating that they were able to rapidly detect spatial and non-spatial changes in their environment.

Taken together, these data demonstrate that the CA3 hippocampal lamination phenotype in *Dcx*-KO mice does not impair the encoding of new experiences when learning required rapid novelty detection and contingent associations.

### Hippocampal Adult Neurogenesis is Normal in *Dcx-KO* Mice

Because the dentate gyrus is a major primary site of continuous adult neurogenesis involved in hippocampal-dependent learning and memory [38], [39], [40], we also quantified proliferation and survival of hippocampal newborn granule cells in adult *Dcx*-KO and WT mice. Our data revealed that *Dcx*-KO mice exhibited similar numbers and spatial distributions of BrdU-labeled proliferating and surviving granule cells as compared to their WT littermates (**Fig. S3**). In addition, to further characterize the phenotype of the newborn granule cells, we quantified the proportion of BrdU-NeuN (neuronal) or BrdU-GFAP (glial) coexpressing cells and found that neuronal as well as glial differentiation were preserved in *Dcx-*KO animals as compared to their WT littermates (59.6% BrdU-NeuN in WT, 48.7% in KO; BrdU-GFAP: 2% WT and 2.5% KO). Altogether, these results indicate that *Dcx* deletion and CA3 lamination defects in this model do not affect hippocampal adult neurogenesis.

### Social Deprivation Altered Social Interaction and Reduced Aggressive Behaviors in *Dcx-KO* Males

Neuropsychiatric disorders and intellectual disability share a core of neurobehavioral deficits characterized by impaired social interactions and deficits in communication. In addition, deficits in social behavior have occasionally been linked to hippocampal dysfunction in both rats and mice [41], [42]. To further characterize *Dcx*-KO mice, we first verified that both mutant and WT males were able to discriminate between male and female scented sawdust and they all spent more time sniffing the male odors compared to the female odors (Fig. S4A). We then used a modified version of a three-chamber social arena [43] to probe animals for their voluntary initiation of social interaction and their ability to discriminate social novelty. We did not detect any significant difference in sociability between *Dcx*-KO and WT males that all spent more time in close proximity with an unfamiliar (stranger) male compared to a cage mate (familiar) male (Fig. S4B). Altogether, these data revealed no obvious deficit in olfactory acuity and normal social memory in *Dcx-*KO mice. These observations are further strengthened by data obtained in an olfactory reward task, showing that odor discrimination is not affected in *Dcx*-KO mice on the B6×129F1 hybrid genetic background (Fig. S5).

**Figure 5:**
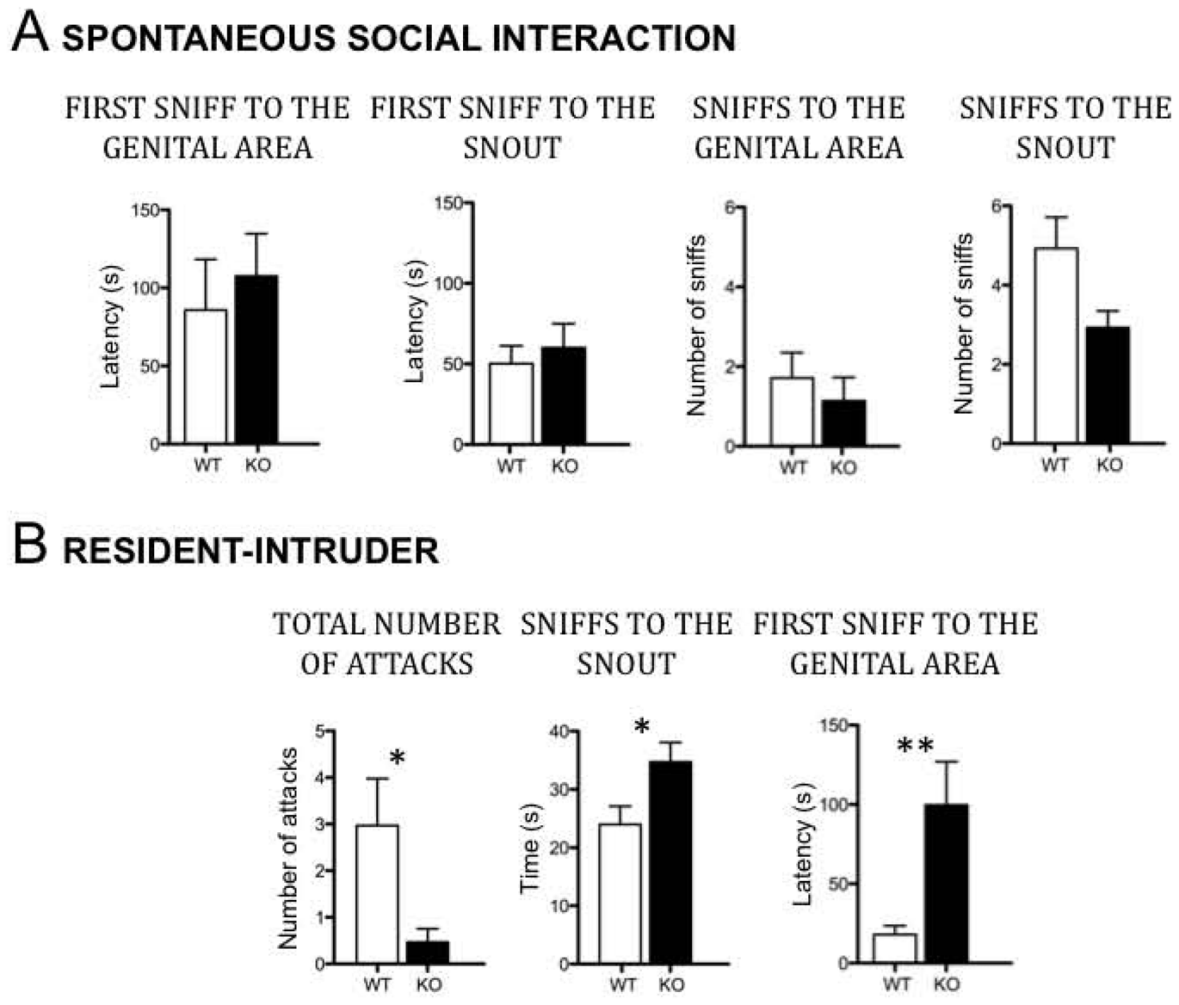
Abnormal social interaction and reduced aggressive behavior in the *Dcx-*KO mice. A/Spontaneous social interaction *;* Performances of *Dcx*-KO male mice (n = 7 mice per genotype) expressed as the latency of the first sniff to the genital area or snout, and the total amount of time spent sniffing the genital area or the snout, measured over a 5-min period. **B/Resident-Intruder** *;* Performances of *Dcx*-KO (n = 17) and WT (n = 16) male mice in the resident-intruder test expressed the number of attacks, the total amount of time spent sniffing the snout, and the latency of the first anogenital sniff, and, measured over a 10-min period. Values represent means +/− SEM. *p<0.05, **p<0.01.

Spontaneous social interactions between unrelated adult males were very similar between *Dcx*-KO and WT males. They explored the visitor male (ano-genital and snout areas) within the same latency period, and exhibited the same amount of ano-genital sniffing (Fig. 5A), but *Dcx*-KO males sniffed the snout less often than WT males (F_1,12_ = 5.1; p<0.05). Under these normal grouped breeding conditions, no aggressive behavior was observed. However, as expected, aggressive behaviors in the resident-intruder task were increased in WT males following social deprivation. We found that this increase in aggression was less pronounced in *Dcx*-KO animals compared to WTs. Indeed, only 15.5% of the KO mice attacked the intruder, compared with 50% for the WTs. In addition, the number of attacks was significantly reduced for KO mice (Fig. 5B; F_1,31_ = 6.01, p<0.05). Also the exploration behavior of the *Dcx*-KO residents toward the intruders was very different from that of WT mice. Whereas both genotypes exhibited the same total amount of time sniffing the intruder (data not shown), the *Dcx*-KO males spent longer times engaging in nose-to-nose interactions, with a much longer latency of the first sniff to the genital area of the intruder, compared to their WT littermates (respectively, F_1,31_ = 5.5, p<0.05 and F_1,29_ = 9.20, p<0.01). Thus, *Dcx*-KO mice exhibit abnormal responses to intruder males.

### Impaired Behavioral Lateralization in *Dcx-KO* Males

Impaired behavioral lateralization and abnormalities in anatomical and physiological asymmetry have been widely documented among neuropsychiatric and intellectual disability patients [44], [45]. Indeed, we previously showed a loss of behavioral lateralization in another intellectual disability mouse model [46].

**Figure 6:**
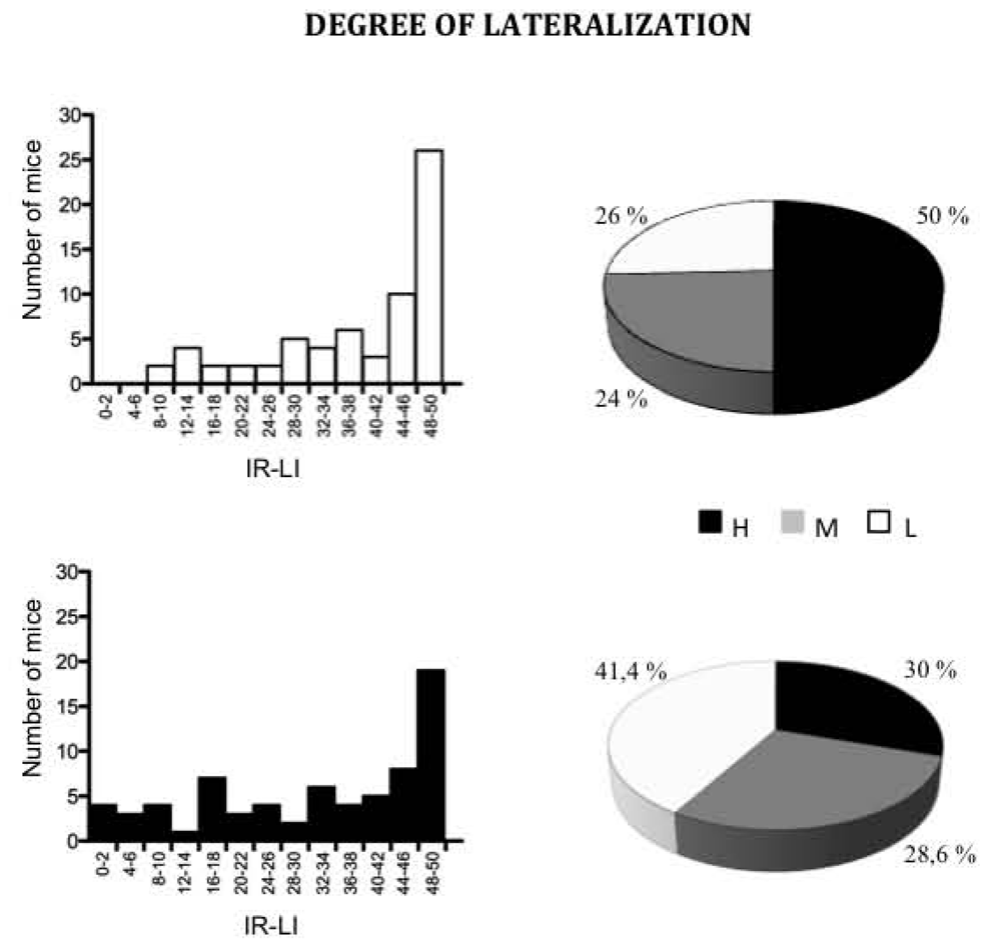
Reduced behavioral lateralization in the *Dcx-*KO mice. Degree of lateralization in *Dcx*-KO (n = 70) and WT (n = 66) male mice. **A/**Distribution for the |R–L| variable that takes even values from 0 to 50*;* **B**/Distributions for the three classes of highly (H) lateralized mice (|R–L| ≥46), ambidextrous (L) mice (|R–L| ≤30), and intermediate (M) mice scoring between 32–44.

We thus assessed paw preference in *Dcx*-KO mice using the Collins paradigm [47]. There were as many right-handed as left-handed subjects in the two genotypes (data not shown). However, among WT males, 50% showed high lateralization, performing more than 47 food reaches with the same paw (out of 50 tested), compared with only 30% of the *Dcx*-KO mice (χ^2^_(2)_ = 9.6; p<0.01), indicating that *Dcx*-KO mice were less strongly lateralized than WT mice (Fig. 6).

## Discussion

*Dcx*-KO mice exhibit an abnormal double layer of hippocampal CA3 pyramidal cells that are characterized by reduced dendritic arbors and an increased excitability [13], [14]. The most striking result of the present study is that in spite of these CA3 anatomical and physiological defects, *Dcx*-KO mice showed normal hippocampal- and CA3-dependent cognitive processes. On the other hand, we report that the *Dcx* mutation is associated with altered social behaviors and a loss of behavioral lateralization, two phenotypes commonly observed in psychiatric disorder and intellectual disability patients [44], [45].

A gradient of severity of cortical abnormalities has been previously reported among the different mouse models of type 1 lissencephaly. This gradient extends from severe and extensive neuronal disorganization in the *reeler* model, to subtle isocortical defects associated with ectopic pyramidal cells in the hippocampus of both the *Lis1* and *Tuba1a* models, and *Dcx-*KO mice, where cellular disorganization is largely restricted to the CA3 region. This gradient apparently parallels the gradient of severity that is observed at the behavioral level. We report here normal hippocampal-dependent memory in our *Dcx*-KO model, whereas both *Tuba1a* and *Lis1* mice exhibited hippocampal-dependent memory deficits, as tested in the T-maze or in the Morris water maze, respectively [4], [48]. Consistent with their extensive neurological disorder, the behavioral phenotype of the *reeler* mice is markedly abnormal, with severe dystonia, ataxia, and tremor, as well as spatial working memory defects and other social and emotional behavioral abnormalities [49].

Our data, showing no hippocampal-related behavioral dysfunction in *Dcx*-KO mice, contrast with behavioral deficits observed in the previously reported *Dcx-*KO mouse model, in fear conditioning and water maze tests [11]. In this mouse model, *Dcx-*KO males showed early postnatal lethality and behavioral tests were conducted on heterozygous *Dcx* females. This latter *Dcx* mutation was maintained on either a mixed (NIH black Swiss and 129/SvJ) or an inbred (129/SvJ) genetic background different from the B6 background used to maintain our mice, which could contribute to the differences between the two studies. Indeed, we have previously reported anatomical differences between the B6 and Sv129*Pas* backgrounds [1]. However, we never observed premature death of *Dcx-*KO males maintained either on the B6, Sv129*Pas*, or an F_1_ hybrid background. Thus, our *Dcx*-KO model, although largely identical anatomically, differs at the behavioral and viability levels from the previously described model, perhaps due to key modifier gene differences [50].

Our data, showing remarkably preserved hippocampal- and CA3-dependant cognitive function are unexpected. On the one hand, the absence of *Dcx* affects not only migration, but also maturation and excitability of CA3 pyramidal cells of the dorsal hippocampus [13], [14] that is known to be important for spatial navigation [51]. However, on the other hand, more detailed anatomical and physiological data revealed that synaptic targeting by excitatory (mossy fibers) and parvalbumin-positive inhibitory (peri-somatic interneurons) systems on CA3 pyramidal cells seems largely preserved in both CA3 layers of *Dcx*-KO mice, although some mossy fiber innervation abnormalities were observed [14]. These latter data suggest that hippocampal synaptic transmission and plasticity could still be retained to an extent compatible with normal hippocampal-dependent cognitive function. A further electrophysiological characterization of synaptic transmission and plasticity, more specifically focused on CA3 inputs from the perforant pathway, mossy fibers, and recurrent collaterals in *Dcx*-KO mice will further help to clarify these results. It is however, also interesting to note, that phylogenetic differences in lamination have arisen, such that a bi-laminar CA1 region exists naturally in various mammalian species [52]. Bi-lamination *per se*, therefore is not necessarily expected to lead to aberrant functional consequences.

Several compensatory mechanisms may contribute to the absence of obvious hippocampal-dependent cognitive deficits in *Dcx*-KO mice. Some previous studies have clearly shown intact spatial navigation performance despite focal CA3 impairment [18], [27]. More generally, though the hippocampus is known to play an important role in processing and recalling spatial and contextual information, it is also widely accepted that all tasks can be learnt to some extent in the absence of an intact hippocampal system, presumably by other learning/memory systems that remain intact and exert an adaptive function. For example, post-training hippocampal lesion produces severe retrograde amnesia, whereas pre-training lesions do not produce reliable anterograde amnesia, suggesting that in the absence of a functional hippocampus, alternative systems may take over [20], [21]. In addition, because hippocampal lamination defects in *Dcx*-KO mice can be detected as early as E17.5 [1], an adaptation of alternative neuronal circuits may have occurred. Also, in response to germline gene targeting, genetic redundancy may be activated to exert a compensatory function and hence protect the integrity of a system. Double knockout mice inactivated for both *Dcx* and *doublecortin-like kinase1,* and acute gene inactivation, through the use of shRNA against *Dcx*, have already demonstrated partial redundancy for multiple neuronal functions [53], [54]. Such redundancy may also contribute to the limited defects observed at the cognitive level, despite the anatomical abnormalities.

Adult hippocampal neurogenesis and neuron survival were also unaffected in *Dcx*-KO mice. Previous studies showed that *Dcx* inactivation results in a severe migration defect in the rostral migratory stream, stemming from the subventricular zone, a second region of adult neurogenesis, where neurons migrate long distances to the olfactory bulb [12], [55], [56]. Our present data, showing normal adult neurogenesis in the subgranular zone and positioning of newborn neurons in the dentate gyrus, suggest that only long-distance migrating newborn neurons in the adult brain might be susceptible to inactivated *Dcx* [57]. An absence of obvious neurogenesis defects in the *Dcx*-KO adult hippocampus certainly would contribute to its apparently normal functioning at the cognitive level.

We report that the *Dcx* mutation is associated with altered social interaction, reduced aggression, and a loss of behavioral lateralization. It is also noteworthy that in the fear-conditioning paradigm, we incidentally observed that *Dcx*-KO mice always exhibited a small tendency to freeze less than their WT littermates (see Figure 3). This reduced freezing, observed even before the shock, is not memory related, but could be indicative of altered anxiety-related phenotype. A further behavioral characterization of *Dcx*-mutant mice, more specifically focused on stress and anxiety, will help to clarify these results.

Interestingly, similar phenotypes of altered social behaviors and reduced behavioral lateralization were previously reported in other mouse models of intellectual disability such as, for example, *Ophn1* [46], *Gdi1* [69], *Fmr1* [70], *motopsin* [71], and *Pten* [72] mutants. Using magnetic resonance imaging and histological approaches, we previously reported a strong association between mutated *DCX/Dcx* and corpus callosum abnormalities, the severity of which, ranging from complete agenesis to a normal corpus callosum, varies in both human and mouse species depending on the genetic background [1]. Further studies will help to identify the potential contribution of corpus callosal abnormalities to defects of behavioral lateralization.

Altogether, the present results suggest that further anatomical and cellular characterization of *Dcx*-mutant mice, expanding beyond the hippocampus into other regions such as the amygdala and prefrontal cortex, would be pertinent to further understand their social and emotional-related phenotypes.

Finally, in humans, recent studies have shown lissencephaly is also associated with tangential interneuronal migration deficits [58], [59]. Such interneuronopathies, initially described for *ARX/Arx* mutations [60], have also been reported in mouse models for *Lis1* [61], [62], *reeler* [63], and *Dcx* [12], [55], [64], [65]. Interneuron abnormalities are suspected to underlie a variety of neurodevelopmental and psychiatric disorders in humans, including epilepsy, schizophrenia, mood, and autism spectrum disorders [66], [67], [68]. It will thus be important to further explore the potential contribution of these interneuron defects to the behavioral phenotypes reported here for *Dcx* mutants.

More generally, altered social and anxiety behaviors, and abnormal lateralization are major endophenotypes for human neuro-developmental disorders, accessible via rodent models, and may thus contribute to bridge the gap between clinical and experimental settings.

## Materials and Methods

All experiments were conducted in accordance with the European Communities Council Directive (86/609/EEC) regarding the housing, care, and experimental procedures on mice. Authors GG and CD are authorized to conduct animal experiments by the Ministry of Agriculture. MNB holds an authorization to conduct animal experiments from the Direction Départementale des Services Vétérinaires. JG has a certificate to conduct animal experiments from Paris Descartes University. IFR-83 Institute of Integrative Biology UPMC is authorized for animal experiments by the Prefecture de Police in Paris (authorization B 75-05-24). INSERM Unit 513 - Neurobiology and Psychiatry is authorized by the Prefecture of Val de Marne (authorization 94-028-21 A).

### Mice

*Dcx* mutant mice were maintained on the C57BL/6J (B6) background following more than 10 generations of backcrosses. All animals were produced and genotyped as previously described [12].

Animals were weaned at 4 weeks and housed two to four per cage by sex and litter regardless of the genotype under standard conditions, with food and water available *ad libitum*. Experiments were always conducted during the light phase of a 12 h light/dark schedule (lights on at 07∶30 a.m.), in a sound-attenuated room under controlled illumination and by trained observers blind to the genotype.

All behavioral tests were performed on adult (3–7 months of age) *Dcx*-KO males and their WT littermates. For each protocol, 2 to 4 independent experiments with different pools of mice were run in initial investigations to ensure robust data generation. All mice were included in two protocols with a minimum of two weeks between each test, and all mice were tested for behavioral lateralization as a final test. All experiments were conducted in accordance with the European Communities Council Directive (86/609/EEC) regarding the housing, care, and experimental procedures on mice (agreement B 75-05-24).

### Plot Profiles Analysis for Lamination of the Pyramidal Layer in the CA3 Region

In order to characterize the lamination abnormalities in the hippocampus, three sagittal sections covering the medio-lateral extension of the dorsal hippocampus, from 8 WT and 8 *Dcx*-KO animals, were immunostained with NeuN (see below) to be able to visualize pyramidal cell layers. Plot profiles of fluorescence corresponding to the CA3 pyramidal cell layers were generated on these sections using ImageJ (NIH, Bethesda, MD).

### BrdU Labeling, Immunohistochemistry and Neurogenesis Quantification

In order to investigate the effect of *Dcx* mutation on the proliferation of progenitor cells and their later survival in the dentate gyrus of the hippocampus, adult mice were injected intraperitoneally with 5-bromo-2′-deoxyuridine (BrdU; Sigma, St. Louis, MO, 100 mg per kg, IP, dissolved in 0.9% NaCl in H_2_O). Proliferation was examined 2 h after one BrdU injection, whereas survival was tested 28 days following 4 BrdU injections every 2 h for 6 consecutive hours. Animals were deeply anesthetized and perfused transcardially with 4% paraformaldehyde in 0.1 M phosphate buffer saline (PBS). The brains were dissected, postfixed overnight, and sagittal sections (40 µm) of one hemisphere were serially cut using a vibrating microtome (Leica) and collected in PBS with sodium azide 0.02%. Immunohistofluorescence and quantification analysis were performed as described previously [73], [74]. Briefly, every sixth section throughout the hippocampus was stained with an anti-BrdU antibody to assess proliferation, or anti-BrdU and NeuN or GFAP (glial fibrillary acidic protein) antibodies to assess cell survival and neuronal or glial differentiation. Sections were washed with Triton X-100 (0.5% in PBS), denatured in 2 N HCl for 30 min at 37°C, washed three times in PBS, and then blocked in PBS containing 2% milk protein (Regilait). The following primary antibodies were used: rat anti-BrdU (1∶1000; AbCys) and mouse anti-NeuN (1∶100, Chemicon) and mouse anti-GFAP (1/1000, Chemicon). Sections were incubated overnight at RT in primary antibody, washed with PBS, then incubated at room temperature for 90 min with secondary antibody, goat anti-rat Alexa-555 and goat anti-mouse Alexa-488 (1∶400, Invitrogen). Sections were then washed and coverslipped with fluoromount-G (Southern Biotech). In each section, every BrdU-positive cell within the granule cell layer and adjacent subgranular zone, defined as a two-cell-body-wide zone along the border of the granule cell layer, was counted through a 40× objective using a Zeiss (Germany) microscope. Data are presented as absolute numbers of BrdU-labeled cells and were obtained by multiplying BrdU-cell density by the reference volume [73], [74]. Adult *Dcx*-KO (n = 2/10) mice exhibiting an ectopic expression of NPY in the hippocampus and which were thus likely to have had epileptic seizures [13] were not included in our neurogenesis analysis. For double-labeling, percentages of BrdU-labeled nuclei co-expressing NeuN or GFAP were determined by analyzing 50 randomly selected BrdU-labeled nuclei throughout the granule cell layer with an Apotome optical sectioning device (Zeiss, Germany). BrdU-positive nuclei were analyzed (63× oil objective) in their entire *z*-axis (0.5 µm steps) and were rotated in orthogonal planes (*x–y*) to verify double labeling and exclude false double labeling caused by overlay of signals from different cells.

### Barnes Circular Maze

The protocol for spatial learning and memory was adapted from Bach [75]. The Barnes maze consisted of an open circular PVC platform 125 cm in diameter, elevated 46 cm above the floor. Twenty holes, 5 cm in diameter, were equally spaced around the perimeter of the circle. To escape the aversive light (400 lux) and open space, the mouse learns to locate a darkened escape tunnel (15×16.5×4.5 cm) placed underneath one of the holes, using spatial distal cues in the room. Each trial started with the mouse placed in the middle of the maze under a start chamber for 10 s and ended when the mouse entered the goal tunnel or after 3 min had elapsed. Animals that did not succeed were gently guided to the tunnel and allowed to remain in it for 30 s. In order to avoid olfactory cues, the platform and the tunnel were cleaned between each animal, the platform was rotated 60° and a new tunnel was placed under a new hole, at the same spatial position related to the spatial cues. The mice were trained for 5 consecutive days with 4 trials per day and 15 min inter trial interval (ITI). At the end of the training phase, mice were given a quadrant test in which the tunnel was removed and the strategy to search the tunnel was recorded for 3 consecutive minutes. A video tracking system (View Point, France) was used to monitor variables such as distance and latency to escape, speed, and errors (defined as searches of any hole that did not lead to the tunnel).

### Fear Conditioning

#### Apparatus

Conditioning was performed in a standard mouse rectangular dark chamber made of opaque walls with a stainless steel rod floor. The rods were wired to a shock generator and scrambler for the delivery of foot shock. The chamber was cleaned with 10% detergent solution and a drawer containing clean sawdust was placed underneath the grid floors and changed between each animal. A video camera was positioned at the back of the chamber to allow the subject’s behavior to be observed and recorded by an experimenter in an adjacent room. Memory for either the context-shock or tone-shock association was assessed by measuring the amount of freezing exhibited by the mice when re-exposed to the context or to the tone delivered in a novel context. The novel context consisted of a round Plexiglas box, with a sawdust floor, placed in an illuminated compartment.

#### Contextual and auditory-cue fear conditioning (two pairing trials)

This experiment took place in the conditioning context with the loudspeaker producing the tone (80 dB) located on the top of the cage. Training consisted of a single exposure to the conditioning for 7 minutes, during which two pairs of tone and electrical foot shock were given. Once placed in the chamber, the mice were allowed to explore the context for 3 min and then were exposed to 2 pairings of a tone (1000 Hz, 80 db, 30 s) co-terminating with a foot shock (0.8 mA, 2 s) separated by a 1 min 30 s interval between tone deliveries. One minute and 30 s after the second shock, mice were gently removed from the chamber and returned to their home cage. On the testing day (24 h later), cued memory was tested in the novel context. The amount of freezing was recorded for 60 s before receiving the tone, during the 30 s tone presentation, and for the 90 s after the tone. Mice were then gently removed from the chamber and returned to their home cage. One hour later, mice were placed back into the conditioning chamber and the amount of freezing was recorded during 3 min for contextual memory test.

#### Contextual fear conditioning (one pairing trial)

For one-trial contextual conditioning, mice were placed into the chamber for a 3-min exploration period before the foot shock (1 mA, 2 s) was delivered. Mice were removed from the conditioning chambers one min after shock presentation and returned to their home cage. Three hours or 24 hours after the conditioning session, the mice were individually checked for freezing to the context in the conditioning chamber for 3 min.

#### Contextual fear conditioning (immediate shock procedure)

The procedure was the same as before (one trial, 1 mA, 2 s shock) except that once the mice were placed into the chamber, the placement-to-shock interval (PSI) was reduced to 30 or 40 s and mice were removed from the conditioning chamber 30 s after the shock. Twenty-four hours after the conditioning session, the mice were individually checked for freezing to the context in the conditioning chamber for 3 min.

### Paired Associate Learning

In this task, learning requires distinguishing between two environmental contexts and associating each context with the correct rewarded olfactory cue. The same olfactory cues are used in both contexts, but the reward assignment of each odor is contingent on the context. The protocol was adapted from [36]. During shaping and testing, mice were maintained at 85+/−5% of their free-feeding weight and were permitted *ad libitum* access to water.

The two contexts (CX1 and CX2) consisted in standard clear Plexiglas cages (33×21×18 cm) contrasted by their position in the room, the amount of light, the texture of the floor, and the wall decoration. Each context contained three opaque plastic cups (6 cm in diameter and 5 cm high), which were filled with different scented-sawdust. Each scented-sawdust was prepared with 3 g of an odor (onion, coffee, mint, vanilla tea, cinnamon, or thyme) for 100 g of sawdust and 3 g of crushed reward pellets (Chocapic, Nestle) to mask the scent of the reward that consisted of a small piece of chocolate hidden at the bottom of one of the cups. In CX1, only the cup with A-scented sawdust was baited, whereas in CX2, only the cup with B-scented sawdust was baited. The position of the cups with respect to each other was pseudorandom.

During the 5 days of the *Shaping phase,* animals were trained to dig in a cup to get a reward. On the first day, each fasting mouse was exposed to one cup with chocolate treats dispersed on the surface of the sawdust. On the second and third days, the same procedure was repeated three times, except that the mice were exposed simultaneously to two cups, with the treat hidden in the sawdust of one cup only. On days 4 and 5, mice were given only 8 min to retrieve the treat. After 8 min, if the mouse did not dig, a treat was presented on the surface of the sawdust.

During the 5 days of *Paired associate learning*, each mouse underwent eight trials per day, four in each context, following a pseudorandom order constrained by not having more than two trials in the same context consecutively and counterbalanced for position of the baited cup. The ITI was 15 min. Each trial started by placing the mouse in a context and ended when the mouse retrieved the treat. The response was considered correct if the mouse started digging in the baited cup. Note that if the mouse started digging first in the nonbaited cup, it was allowed to dig in the other cups to correct itself, and the response was considered incorrect.

In order to test their capacities of rapid learning novel contingent associations, all animals were then tested in new contexts (CX3 and CX4) located in another room with three novel odors for two days.

### Spatial and Novel Object Detection

Mice were tested successively for the detection of novel spatial position of an object and of a novel visual object. The apparatus consisted of a square arena (43×43×25 cm). Mice were given a 10 min habituation session to this arena twice a day for three consecutive days. On the testing day, mice were placed at the center of the arena and were allowed to explore three identical objects during 4 acquisition trials (5 min) separated by a 3 min ITI. On the fifth trial, 3 min later, mice were placed back into the same arena, but one of the objects was moved to a new spatial position (spatial detection). The mice were reexposed one more time to this new configuration of objects and on the seventh trial one object was substituted by a novel object with a different shape (object detection).

Time of exploration of each object, when mice sniffed, pawed at, or looked at an object from a distance of under 1 cm, was quantified in order to calculate the detection using the percentage of exploration time of the displaced object or novel object against the total amount of exploration time.

### Social Interaction

Spontaneous social interactions were tested between two unacquainted same-weight adult males. Males used as visitors were all WT individuals of a mixed genetic background. Both the experimental and the visitor males were introduced simultaneously in a new transparent Plexiglas cage containing fresh bedding and left freely interacting for 5 min. The latency and number of social contacts initiated by the experimental male (sniffing to the genital organs or to the head) were recorded simultaneously by two experimenters blind to the genotype.

In the resident-intruder test, social deprivation is used to increase aggressive behaviors. Male mice were thus housed individually for 10 days before the test and used as residents. C3H naive male mice, housed in groups of 8–9, were used as passive intruders. After transfer of the intruder to the home cage of the resident, the number and cumulative duration of aggressive (biting attacks and tail rattling) and social (sniffing to the genital organs or to the head) behaviors was recorded over a 10 min period simultaneously by two experimenters blind to the genotype.

### Behavioral Lateralization

Adult mice were tested for behavioral lateralization as described previously [76]. In short, animals were deprived of food for 24 hours and then placed individually in a transparent testing chamber. Small pieces of regular food were introduced in a tube perpendicular to the front of the chamber and equally accessible using the right or left paw. Mice were observed individually for a total of 50 consecutive reaches for food. Scores for the degree of handedness were given by the absolute value of the difference between the number of entries with right and left paws noted |R-L| [47]. Animals with |R-L| scores between 46 and 50 are defined as strongly lateralized (High, H), whereas ambidextrous (Low, L) individuals have |R-L| scores ≤30, and ambiguous are intermediate (Medium, M).

### Statistical Analysis

Data were subjected to factorial analysis of variance (ANOVA), to assess the interaction between genotype (between factor) and time (within factor). Significant major effects were analyzed further by *post hoc* comparisons of means using the Newman–Keuls test. For the degree of lateralization, proportions of strongly lateralized, ambiguous, and ambidextrous animals were compared using the χ^2^ test. The significance was established at a *p-*value *<*0.05.

## Supporting Information

Additional Supporting information may be found in the online version of this article:

Figure S1 **Comparison of body weight growth rate between WT and** ***Dcx*** **-KO males.** (n = 50–61 mice per genotype and per age, except at 2 months of age where only n = 5 animals per genotype were available), between 1 and 7 months of age (genotype: F_1, 288_ = 11.25, p<0.001; age: F_1, 6_ = 536.5, p<0.001).
(TIFF)

Figure S2 Comparison of spontaneous locomotor activity between WT and *Dcx*-KO mice in the open-field (n = 16 mice per genotype) and Y mazes (n = 6–8 mice per genotype).
(TIFF)

Figure S3 **Top.** Adult hippocampal neurogenesis is not affected in *Dcx*-KO mice. (A–D) Two hours after a single BrdU injection, the number of BrdU proliferating cells appears similar in controls (A, C) and *Dcx*-KO (B, D). The combination of BrdU staining with NeuN shows that the distribution of newborn cells appears similar in the KO mice. (E–H) Four weeks after the last injection of BrdU, surviving cells are able to migrate a short distance in the granule cell layer in both WT (G) and KO (H) mice. Scale bar 50 µm. **Bottom.** Quantitative comparison of proliferation and later survival of newborn BrdU-labeled cells in the DG (one hemisphere) between WT (n = 10) and *Dcx*-KO (n = 8) males.
(TIFF)

Figure S4 **Comparison of social discrimination performances between WT and** ***Dcx*** **-KO males, measured by (A) the time spent exploring male and females odors (n = 6 WT and n = 8** ***Dcx*** **-KO males) and (B) the time spent exploring familiar and unfamiliar males (n = 16 per genotype).**
(TIFF)

Figure S5 **Odor discrimination is not affected in F_1_- KO (C57BL/6/Sv129Pas).** **A** Schematic drawing of the go/no-go paradigm [77]. A trial is initiated when the animal breaks the infrared (IR) beam (black dashed line) by entering his snout into the sampling port (1). After 1 s, the odor stimulus is presented into the odor sampling port (2). For S+ odor, animals get a water reward if they lick the water tube during the 2 s of odor presentation (3). For S- odor, the animal retracts his head from the sampling port (3′). The percentage of appropriate responses was determined for each block of 20 trials. A score of 85% implied that mice had correctly learned to assign reward/non reward values. Each mouse underwent a session of 10 blocks (200 trials) per day. For each block, the mean behavioral performance (percentage of correct responses) was calculated for each group. **B** Mean percentage of correct responses for different monomolecular odor pairs (indicated in the top part of the panel) for WT (black bold line, n = 8) and *Dcx*-KO mice (dashed line, n = 7). For carvone odor, S+ is (+)-carvone and S- correspond to (−)-carvone (« 100/0 ») or to a mix of (−)-carvone and (+)-carvone (« 2/98 » being 2% of (−)-carvone with 98% of (+)-carvone).
(TIFF)
