# Supplementary figures and images for: Doublecortin Knockout Mice Show Normal Hippocampal-Dependent Memory Despite CA3 Lamination Defects
Source: PLoS One. 2013 Sep 20;8(9):e74992. doi: 10.1371/journal.pone.0074992 (PMC3779246; doi:10.1371/journal.pone.0074992)

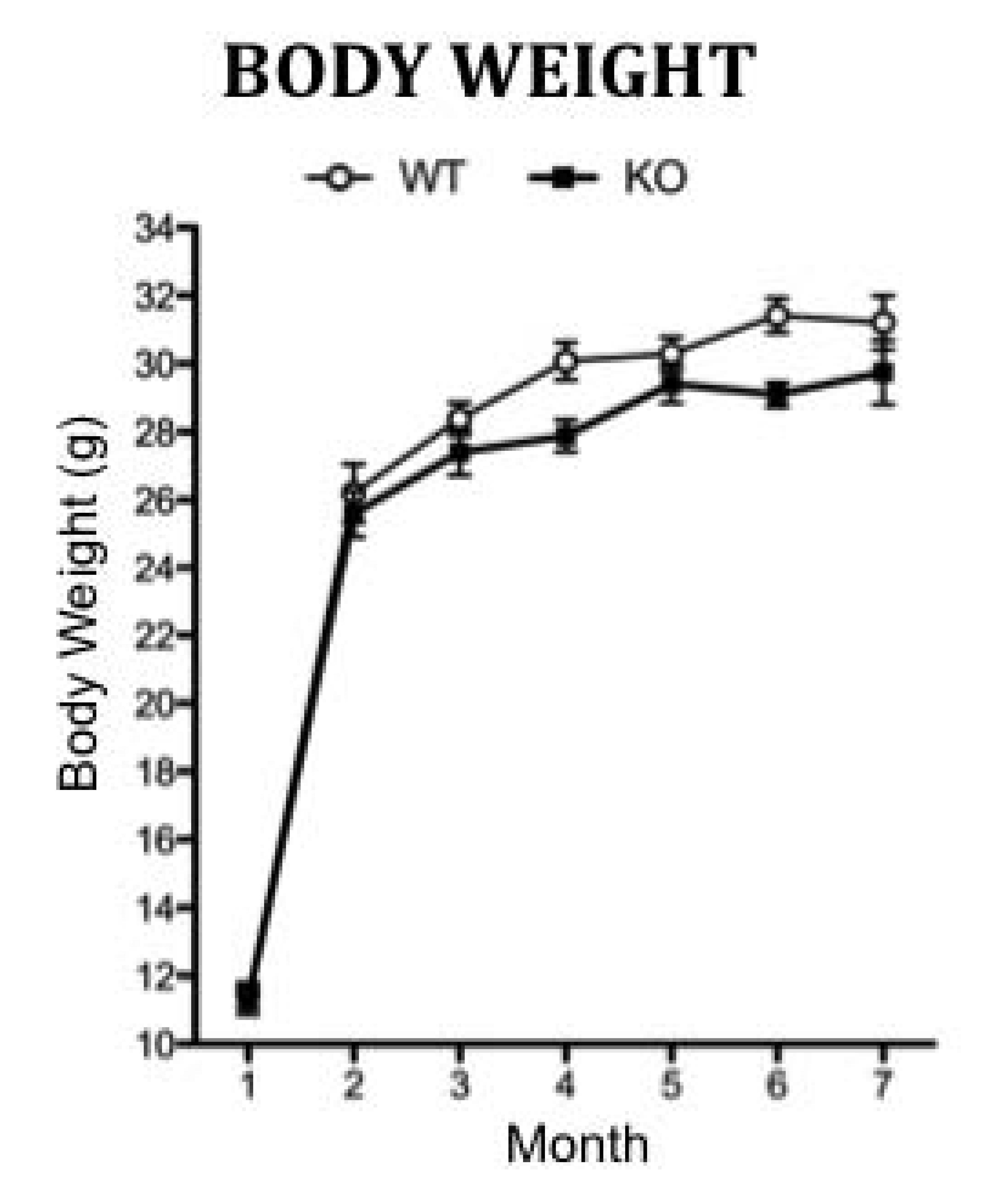

Supplement: Figure S1 — Comparison of body weight growth rate between WT and Dcx -KO males. (n = 50–61 mice per genotype and per age, except at 2 months of age where only n = 5 animals per genotype were available), between 1 and 7 months of age (genotype: F1, 288 = 11.25, p<0.001; age: F1, 6 = 536.5, p<0.001). (TIFF) [file pone.0074992.s001.tiff]

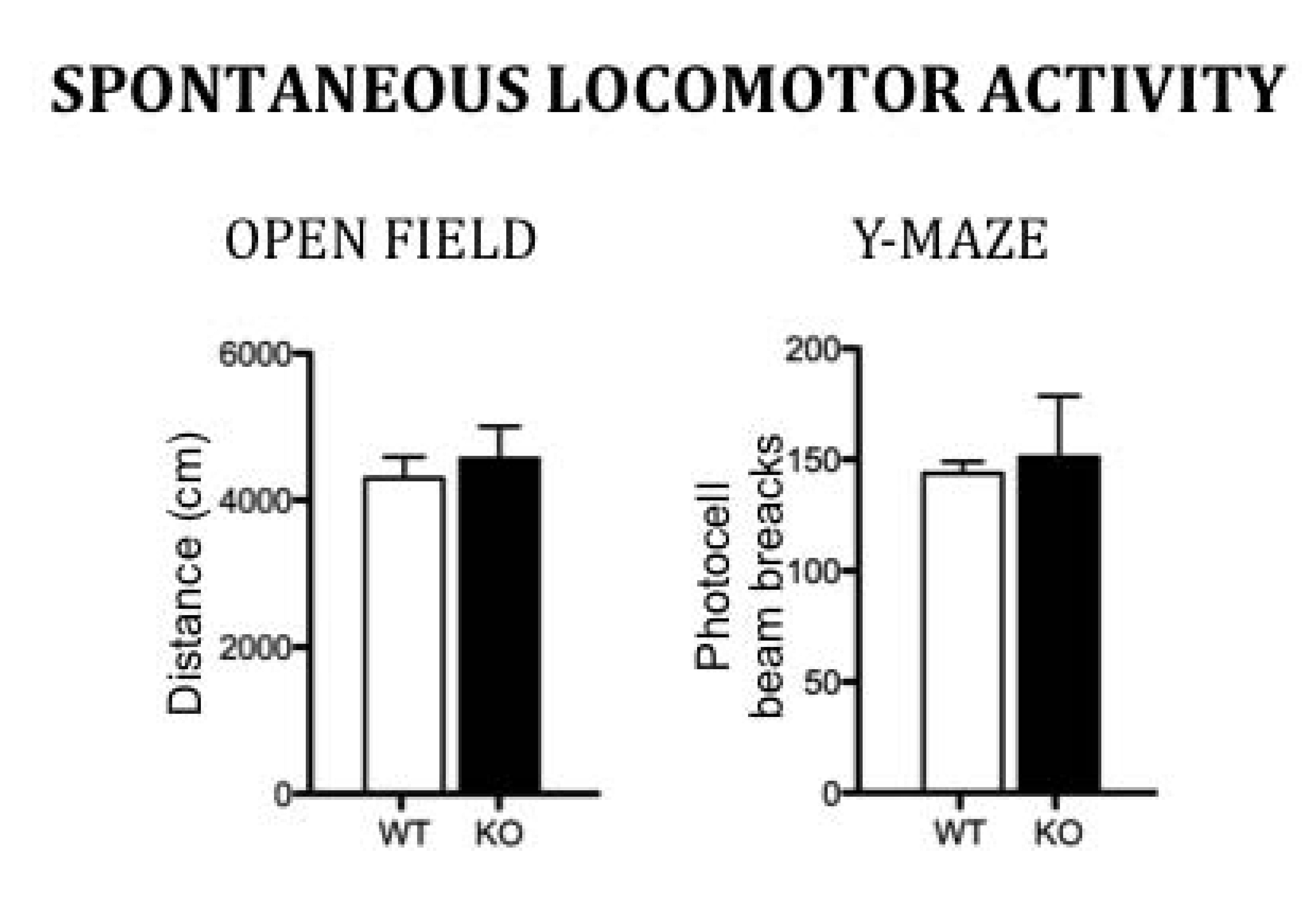

Supplement: Figure S2 — Comparison of spontaneous locomotor activity between WT and Dcx-KO mice in the open-field (n = 16 mice per genotype) and Y mazes (n = 6–8 mice per genotype). (TIFF) [file pone.0074992.s002.tiff]

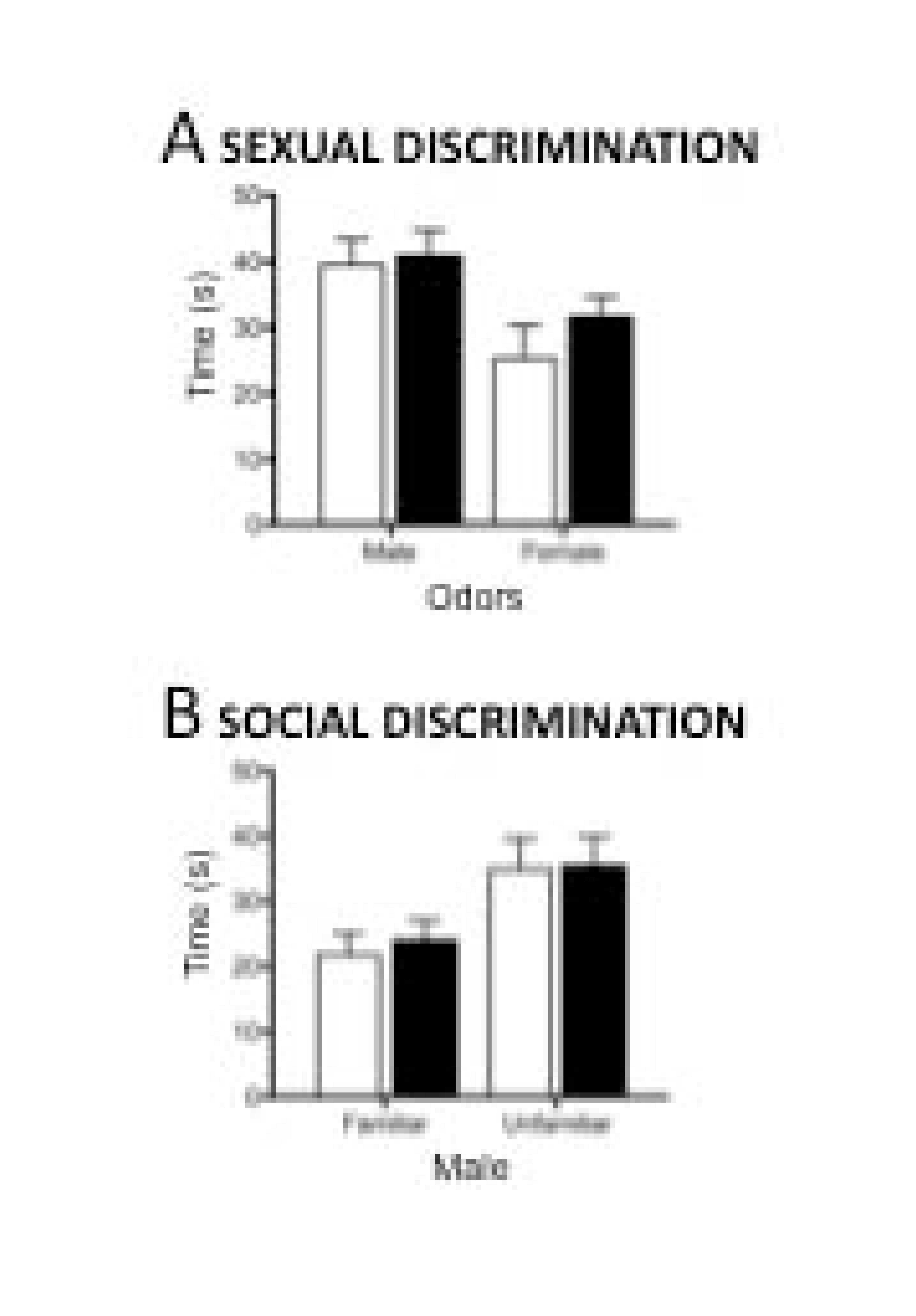

Supplement: Figure S4 — Comparison of social discrimination performances between WT and Dcx -KO males, measured by (A) the time spent exploring male and females odors (n = 6 WT and n = 8 Dcx -KO males) and (B) the time spent exploring familiar and unfamiliar males (n = 16 per genotype). (TIFF) [file pone.0074992.s004.tiff]

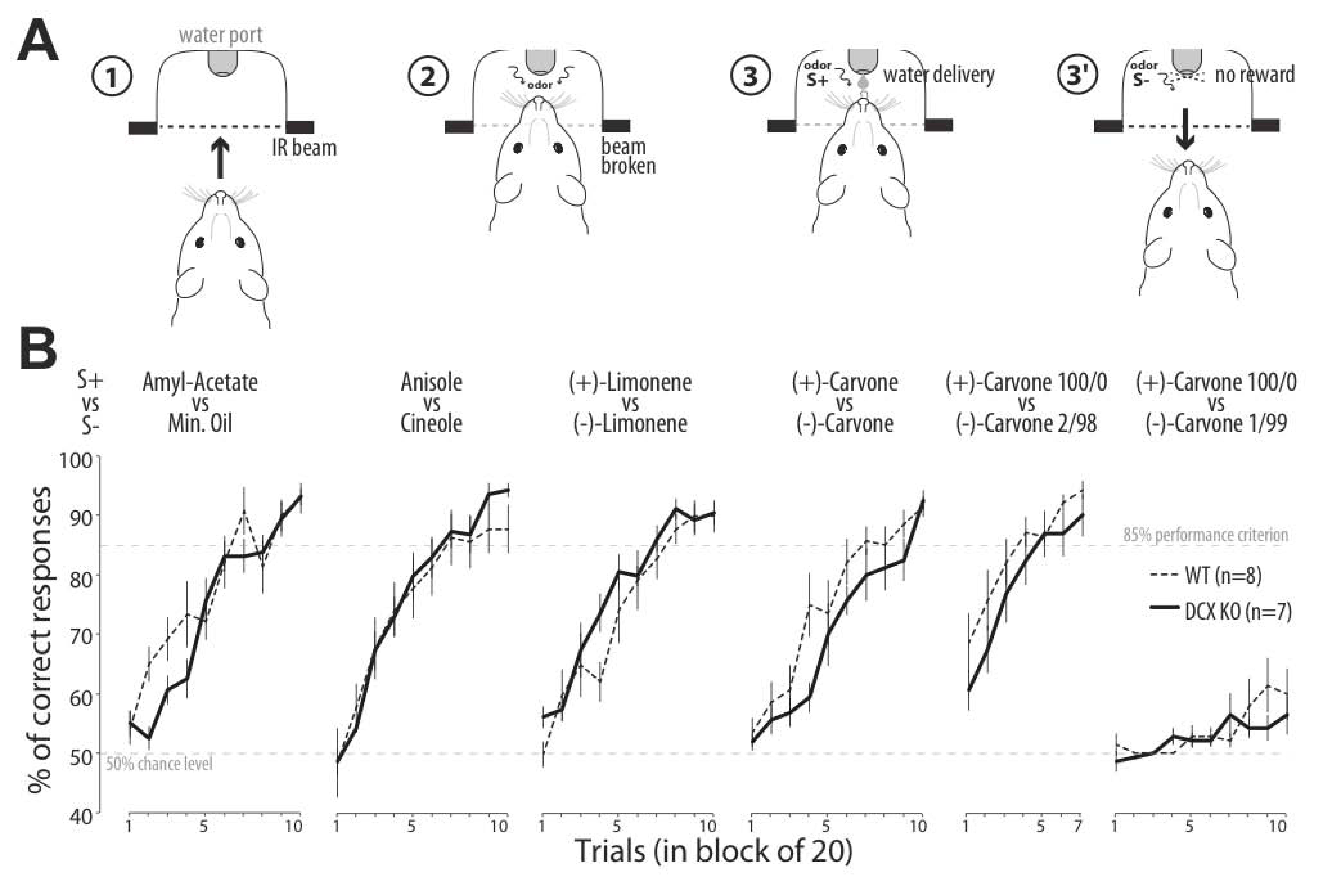

Supplement: Figure S5 — Odor discrimination is not affected in F1- KO (C57BL/6/Sv129Pas). A Schematic drawing of the go/no-go paradigm [77]. A trial is initiated when the animal breaks the infrared (IR) beam (black dashed line) by entering his snout into the sampling port (1). After 1 s, the odor stimulus is presented into the odor sampling port (2). For S+ odor, animals get a water reward if they lick the water tube during the 2 s of odor presentation (3). For S- odor, the animal retracts his head from the sampling port (3′). The percentage of appropriate responses was determined for each block of 20 trials. A score of 85% implied that mice had correctly learned to assign reward/non reward values. Each mouse underwent a session of 10 blocks (200 trials) per day. For each block, the mean behavioral performance (percentage of correct responses) was calculated for each group. B Mean percentage of correct responses for different monomolecular odor pairs (indicated in the top part of the panel) for WT (black bold line, n = 8) and Dcx-KO mice (dashed line, n = 7). For carvone odor, S+ is (+)-carvone and S- correspond to (−)-carvone (« 100/0 ») or to a mix of (−)-carvone and (+)-carvone (« 2/98 » being 2% of (−)-carvone with 98% of (+)-carvone). (TIFF) [file pone.0074992.s005.tiff]
